# Genome-Wide Identification and Expression Profile Analysis of the Phospholipase C Gene Family in Wheat (*Triticum aestivum* L.)

**DOI:** 10.3390/plants9070885

**Published:** 2020-07-13

**Authors:** Xianguo Wang, Yang Liu, Zheng Li, Xiang Gao, Jian Dong, Jiacheng Zhang, Longlong Zhang, Linda S. Thomashow, David M. Weller, Mingming Yang

**Affiliations:** 1 College of Agronomy, Northwest A&F University, Yangling 712100, China; wxianguo1990@sina.com (X.W.); liuy931020@163.com (Y.L.); 15738812341@yeah.net (Z.L.); dj4322@163.com (J.D.); 2 Branch of Bioengineering, Yangling Vocational & Technical College, Yangling 712100, China; zhangjiachengp@163.com; 3 Shangluo Agricultural Science Research Institute, Shangluo 726000, China; zllt2013@163.com; 4 United States Department of Agriculture–Agricultural Research Service, Wheat Health, Genetics and Quality Research Unit, Pullman, WA 99164-6430, USA; thomashow@wsu.edu (L.S.T.); david.weller@usda.gov (D.M.W.)

**Keywords:** wheat, PLC, abiotic stress, expression patterns, *Arabidopsis*

## Abstract

Phospholipid-hydrolyzing enzymes include members of the phospholipase C (PLC) family that play important roles in regulating plant growth and responding to stress. In the present study, a systematic in silico analysis of the wheat *PLC* gene family revealed a total of 26 wheat *PLC* genes (*TaPLC*s). Phylogenetic and sequence alignment analyses divided the wheat PLC genes into 2 subfamilies, *TaPI-PLC* (containing the typical X, Y, and C2 domains) and *TaNPC* (containing a phosphatase domain). *TaPLC* expression patterns differed among tissues, organs, and under abiotic stress conditions. The transcript levels of 8 *TaPLC* genes were validated through qPCR analyses. Most of the *TaPLC* genes were sensitive to salt stress and were up-regulated rapidly, and some were sensitive to low temperatures and drought. Overexpression of *TaPI-PLC1-2B* significantly improved resistance to salt and drought stress in *Arabidopsis*, and the primary root of P1-OE was significantly longer than that of the wild type under stress conditions. Our results not only provide comprehensive information for understanding the *PLC* gene family in wheat, but can also provide a solid foundation for functional characterization of the wheat PLC gene family.

## 1. Introduction

Phospholipids are an important component of cytoplasmic membranes, playing a vital role in plant growth, development, and regulation of responses to abiotic stresses and biotic challenges [1,2]. Phospholipases in plants are responsible for the hydrolysis of phospholipids and are divided into four types: phospholipase A1 (PLA1), phospholipase A2 (PLA2), phospholipase C (PLC), and phospholipase D (PLD). Within each type, there also are subfamilies that differ in structure, substrate, binding site and reaction conditions under which they are active [3]. Based on their substrates, PLCs can be divided into two subfamilies: the phosphatidylinositol specific PLC (PI-PLC) and the nonspecific PLC (NPC) [4,5]. PI-PLC can hydrolyze phosphatidylinositol 4,5-bisphosphate (PIP2) to produce two important signaling molecules, inositol triphosphate (IP_3_) and diacylglycerol (DAG) [6], which release Ca^2+^ from cells and activate members of the protein kinase C (PKC) family, thus participating in plant growth and development [7,8]. Members of the NPC hydrolyze a series of membrane phospholipids such as phosphatidylcholine (PC) and phosphatidylethanolamine (PE) to generate phosphatidylserine (PS), diacetylglycerol (DAG), and the corresponding phosphate groups [9,10,11,12]. When plants NPCs were first discovered, they were not related to any other known members of the plant phospholipase family, but had three conserved regions shared with phospholipase in *Mycobacterium tuberculosis* [12]. Although NPC does not have the C2, X, Y, and EF domains, it contains a phosphatase domain and can hydrolyze phospholipid, so it is classified as a subfamily of the PLCs [6,12,13].

Members of the phospholipase C gene family have been categorized and described in many plant species [3,5,14,15,16] including 9 *PI-PLC* and 6 *NPC* genes in *Arabidopsis thaliana*, 4 *PI-PLC*, and 5 *NPC* genes in rice (*Oryza sativa* L.), 5 *PI-PLC* and 4 *NPC* genes in maize (*Zea mays* L.), and 12 *PI-PLC* and 9 *NPC* genes in cotton (*Gossypium* spp.). In *A. thaliana*, *AtPI-PLC1* played an important role in hyperosmotic stress independent of ABA treatment [17]. *AtPI-PLC2* is involved in seedling growth, the endoplasmic reticulum stress response, and regulation of male and female gametophyte development [18,19], whereas *AtPI-PLC3* and *AtPI-PLC9* contributed to heat resistance [20,21]. Overexpression of *AtPI-PLC5* caused premature leaf decay [22], the *AtNPC4* gene enhanced resistance to high osmotic stress [23]. Abscisic acid induced up-regulation of *AtPI-PLC6* expression and participation in the cold stress response [24]. In *Brassica napus,* overexpression of *BnPI-PLC2* enhanced plant drought tolerance and positively affected phytohormone levels [25,26]. In potato, *StPLC* was involved in regulation of DNA synthesis and the cell cycle [27], and in maize, *ZmPI-PLC1* affected plant development by promoting asymmetric cell division [14].

PLC enzymes also are known to be involved in growth, development and stress responses in wheat (*Triticum aestivum* L.) [5,28,29,30,31]. However, the genes in wheat have not previously been analyzed systematically. In this study, we identified and characterized the wheat PLC gene family, including sequence features, conserved domains, chromosomal locations, phylogenetic relationships, cis-acting elements, tissue specific expression levels, and expression patterns in response to abiotic stresses including low temperature, drought, and salt stress. One of the family members, *TaPI-PLC1-2B*, was demonstrated to play an important role in responding to drought and salt stress. Our study not only presents comprehensive information for understanding the *PLC* gene family in wheat, but can also provide a solid foundation for the functional characterization of the wheat PLC gene family.

## 2. Results

### 2.1. Identification and Analysis of Phospholipase C (PLC) Gene Family Members in Wheat

Following analysis of the PLAZA protein database with HMM and confirmation of the conserved domains by CDD, Pfam, and SMART search tools, a total of 26 wheat *PLC* genes were identified and named TaPI-PLC1-2A to TaNPC7-4A, based on their subfamily, chromosomal position, and genomic homology. Results of analysis by ExPasy indicated that the physicochemical properties of the two groups of encoded proteins differed (Table 1). TaPI-PLC proteins comprised of 585–633 amino acids with molecular weights of 65.7–71.1 kDa and a slightly acidic isoelectric point of less than 7. In contrast, TaNPC proteins were smaller, only 513-554 amino acids in length, with molecular weights of 56.7–61.7 kDa. Seven TaNPC proteins had isoelectric points less than 7, while the other 8 all had isoelectric points greater than 7 and were slightly alkaline. The GRAVY values of TaPI-PLC and TaNPC proteins were less than 0, indicating that members of both families were hydrophilic.

### 2.2. Exon–Intron Structure and Conserved Motifs of TaPLC Genes 

**Figure 1:**
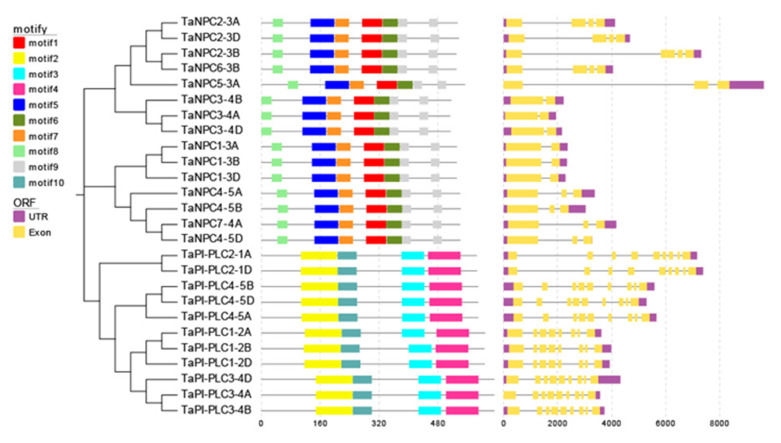
Evolutionary relationship (left), sequence prediction (center), and gene structure (right) of *TaPLC* genes. The evolutionary history was inferred by using the Neighbor-joining method in MEGA7, and the bootstrap value was 1000. The combination of motifs associated with each TaPLC protein is shown in the middle. Ten motifs are marked with boxes of different colors. The EvolView online tool was used to compare the predicted coding sequence (CDS) with the corresponding genomic sequence to detect the exon/intron distribution of the corresponding *TaPLC* gene. The yellow box represents an exon and the gray solid line represents an intron.

**Figure 2:**
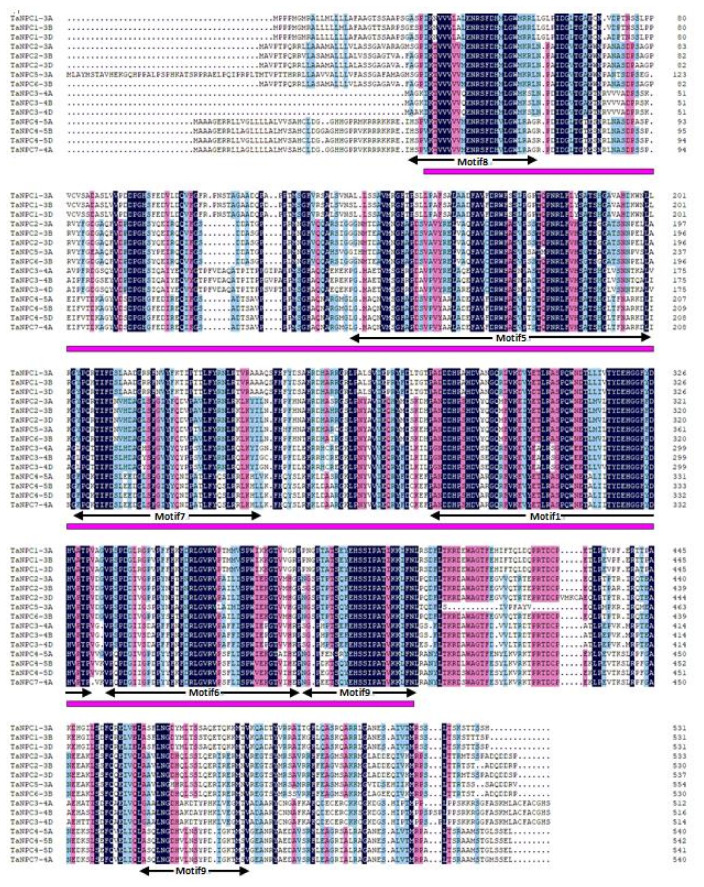
Sequence alignment of the 15 members of wheat NPC proteins. The black lines indicate motifs 1, 5, 6, 7, 8, and 9. The purple lines denote the NPC domain.

**Figure 3:**
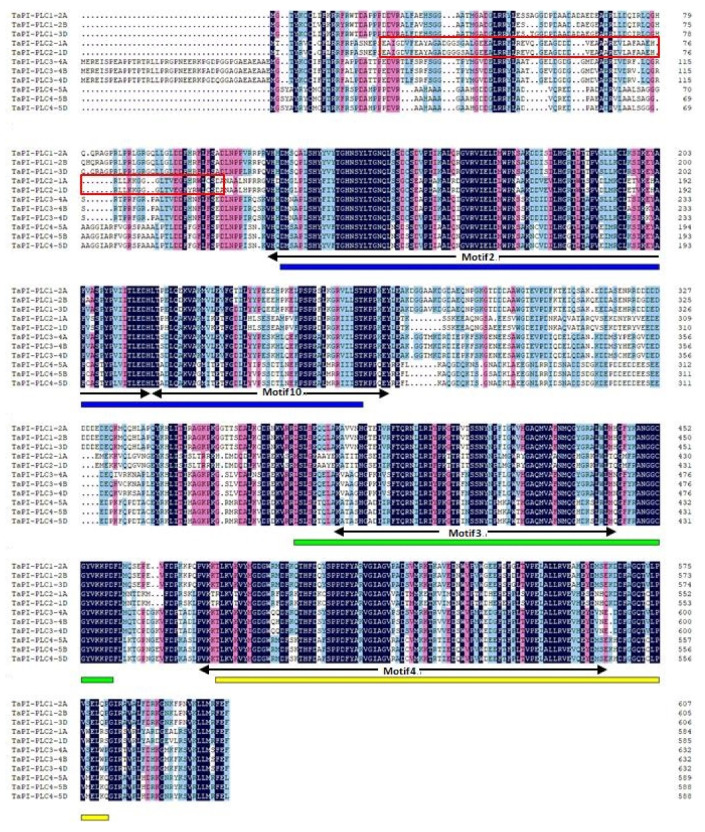
Sequence alignment of the 11 members of wheat PI-PLC proteins. The black lines indicate motifs 2, 3, 4, and 10. The red box represents the EF-hand. The blue, green, and yellow lines represent PI-PLC-X, PI-PLC-Y, and PI-PLC-C2 domains, respectively.

As shown in Figure 1, there are structural differences among *TaPLC* genes, but genes clustered on a branch have similar exon-intron structures, numbers, and distributions of functional motifs. *TaPI-PLC* genes share 4 motifs designated 2, 3, 4 and 10. Except for *TaPI-PLC1-2A*, *TaPI-PLC1-2B*, and *TaPI-PLC1-2D,* which have 8 exons and 7 introns, all the others have 9 exons and 8 introns. The *TaNPC* genes mainly contain 6 motifs including 1, 5, 6, 7, 8, and 9. *TaNPC6-3B* and the three homologous copies of *TaNPC2* contain 4 exons; *TaNPC7-4A*, *TaNPC5-3A*, and the three homologous copies of *TaNPC4* contain 3 exons, while the homologous copies of *TaNPC1* and *TaNPC2* have only two exons. From the protein sequence alignment results, it can be found that motifs 1, 5, 6, 7, 8, and 9 together constitute the phospholipase domain of NPC (Figure 2). While motifs 2 and 10 form the PI-PLC-X domain, motifs 3 and 4 correspond to PI-PLC-Y and PI-PLC-C2, respectively (Figure 3).

### 2.3. Phylogenetic Analysis of TaPLC Genes

**Figure 4:**
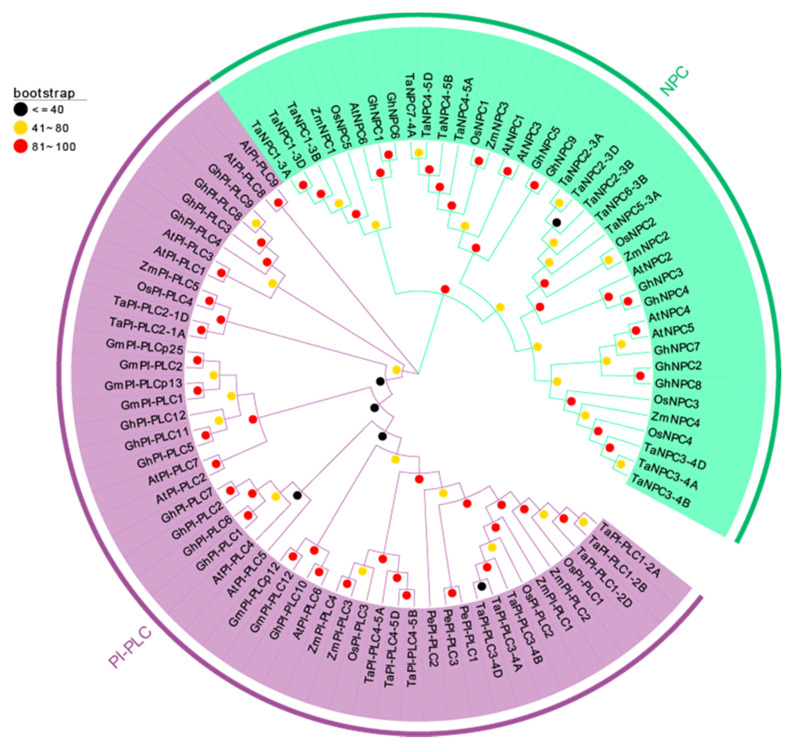
Phylogenetic tree of TaPLCs from wheat, rice, maize, orchid, *Arabidopsis*, soybean, and cotton. The phylogenetic tree was constructed by using the Neighbor-joining method with 1000 bootstrap replications. The PI-PLC and NPC subfamilies are shown in purple and green colors.

In order to precisely reveal the evolutionary relationships of the TaPLC proteins, we performed phylogenetic analyses of 4 monocotyledons (wheat, rice, maize, and orchid) and 3 dicotyledons (*Arabidopsis*, soybean, and cotton) using a neighbor-joining method (Figure 4, Table S1). From the results we can see that the phylogenetic tree showed that 89 *PLC* genes were divided into *PI-PLC* and *NPC* groups totaling 50 *PI-PLC* genes and 39 *NPC* genes, respectively. Genes from monocots and dicots were relatively distantly related, wheat and rice were evolutionarily closer to each other than wheat and maize or wheat and orchid, and *Arabidopsis* PLCs showed high homology to PI-PLCs from soybean and cotton. Similar trends were found for the TaNPC proteins, which more closely resembled those in rice and maize. 

### 2.4. Chromosome Localization, Gene Duplication, and Collinearity Analysis of TaPLC Genes

**Figure 5:**
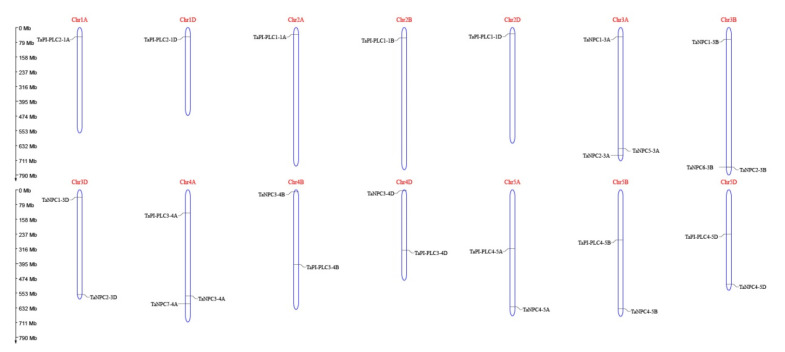
Chromosomal distribution of *TaPLC* genes in wheat. The chromosomal position of each *TaPLC* was mapped according to the physical positions of wheat genomes (Table 1). The chromosome number is labeled at the top of each chromosome. The scale is in mega bases (Mb).

The 26 members of the *TaPLC* family are randomly distributed on 14 chromosomes of wheat (Figure 5), among which the 3A, 3B, and 4A chromosomes carried the most *TaPLC* genes. Chromosomes 1A, 1D, 2A, 2B, and 2D contained fewer members, with only one gene per chromosome. Among the 26 *TaPLC* genes, there were 10, 8, and 8 members distributed on wheat sub-genomes A, B, and D, respectively.

**Figure 6:**
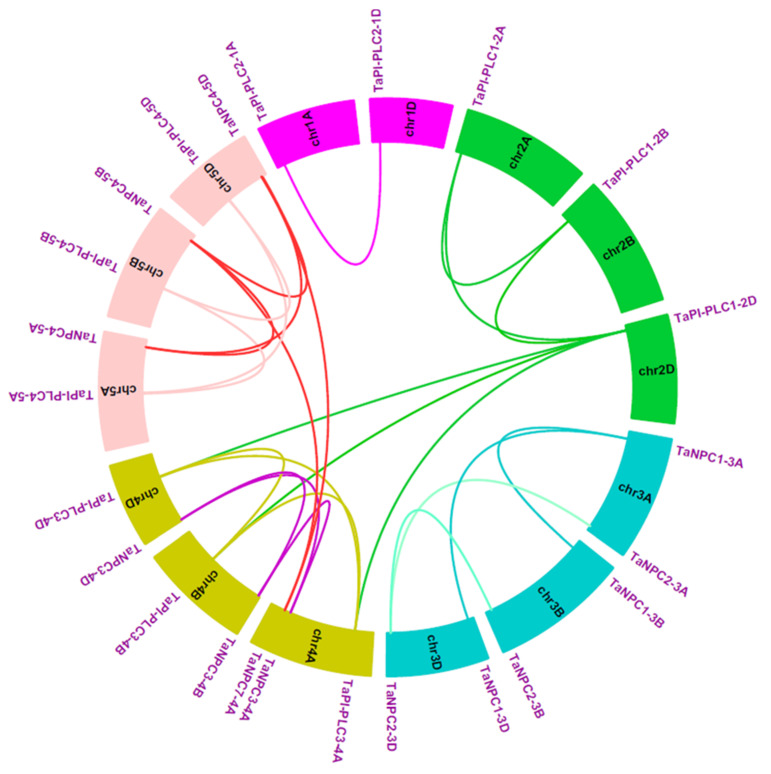
Schematic diagram of the homologous *TaPLC* genes in wheat A, B and D sub-genomes and the duplicated genes pairs identified in wheat.

In terms of gene duplication, there were 7 *TaPLC* members (*TaPI-PLC1*, *3*, *4*, and *TaNPC1*, *2*, *3*, *4*) containing three copies and only *TaPI-PLC2* containing two copies, *TaNPC5-3A*, *TaNPC6-3B*, and *TaNPC7-4A* had only one copy each, which was on chromosomes 3A, 3B, and 4A (Figure 6). Interestingly, *TaNPC7-4A* had the greatest sequence similarity to *TaNPC4-5A* (93.67%), *TaNPC4-5B* (96.13%), and *TaNPC4-5D* (96.31%). Therefore, *TaNPC7-4A* was identified as a segmental duplication gene. *TaNPC5-3A* had the greatest sequence similarity to *TaNPC2-3A* (88.94%), and *TaNPC6-3B* had the greatest sequence similarity to *TaNPC2-3B* (95.17%). Because the genes were distantly located, they were also identified as segmental duplication gene. Therefore, *TaNPC7-4A*, *TaNPC5-3A*, and *TaNPC6-3B* were segmental duplications, and no tandem duplication events involving *TaPLC*s were discovered in the wheat genome. In addition, we found that *TaPI-PLC3-4A* and *TaNPC3-4A* on chromosome 4A were reversed with their homologous genes on chromosomes 4B and 4D (Figure 6).

To better understand the evolutionary factors that affect the *TaPLC* gene family, we calculated the Ka (Nonsynonymous) and Ks (Synonymous) ratios between *TaPLC* gene pairs (Table S2). The Ka/Ks values of the segmentally duplicated *TaPLC* gene pairs, as well as of the orthologous *TaPLC* gene pairs were less than 1, suggesting that members of this gene family might have undergone strong purifying selective pressure during evolution in wheat.

**Figure 7:**
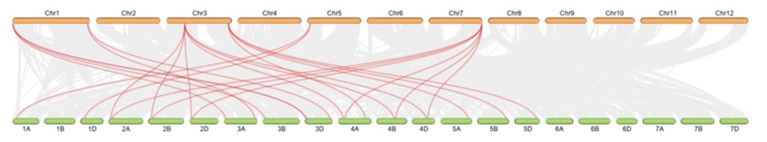
Syntenic relationships between *TaPLC* genes in wheat and rice. Gray lines in the background indicate the collinear blocks within the wheat and rice genomes, while the red lines highlight the synteny of *TaPLC* gene pairs.

Next, synteny analysis between wheat and rice was conducted (Figure 7, Table S3), and 23 orthologous *PLC* gene pairs were found, reinforcing the strong relationship between these plant species revealed by phylogenetic analyses. These results indicated that there was a strong genetic relationship between *PLC* genes in monocotyledons.

### 2.5. Cis-Regulatory Elements in the Promoters of Wheat PLC Genes

**Figure 8:**
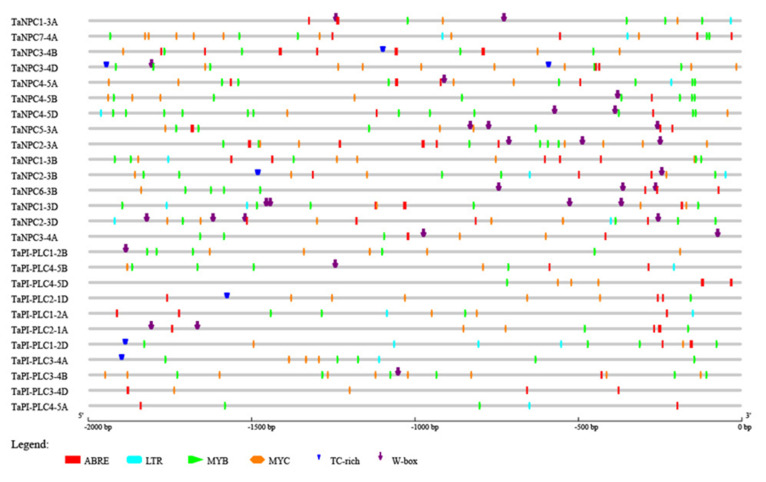
Predicted *cis*-acting elements in *TaPLC* promoters. Promoter sequences (–2000 bp) of 26 *TaPLC* genes analyzed by PlantCARE.

To further evaluate the mechanism of *TaPLC* gene regulation in the abiotic stress response, the 2.0 kb upstream sequences from the translation initiation sites of *TaPLC* genes were submitted to PlantCARE for detection of *cis*-acting elements. Six such elements related to abiotic stress were evaluated including ABRE (Involved in the abscisic acid responsiveness), W-box (involved in dehydration responsiveness), MYB (Involved in drought-inducibility), MYC (involved in the drought and abscisic acid responsiveness), LTR (involved in low-temperature responsiveness), and TC-rich repeats (involved in defense and stress responsiveness) (Figure 8). Each *TaPLC* gene contained at least two stress-related *cis*-acting elements, with ABRE, MYC, and MYB found in almost all *TaPLCs.* From 1 to 3 LTRs were present in 13 *TaPLCs*, and TC-rich repeats and W-boxes were located in 6 and 16 *TaPLCs*, respectively. These results indicated that the individual *TaPLC* genes can respond to multiple abiotic stresses.

### 2.6. Expression Patterns of TaPLC Genes in Different Tissues, Organs, and Stresses

**Figure 9:**
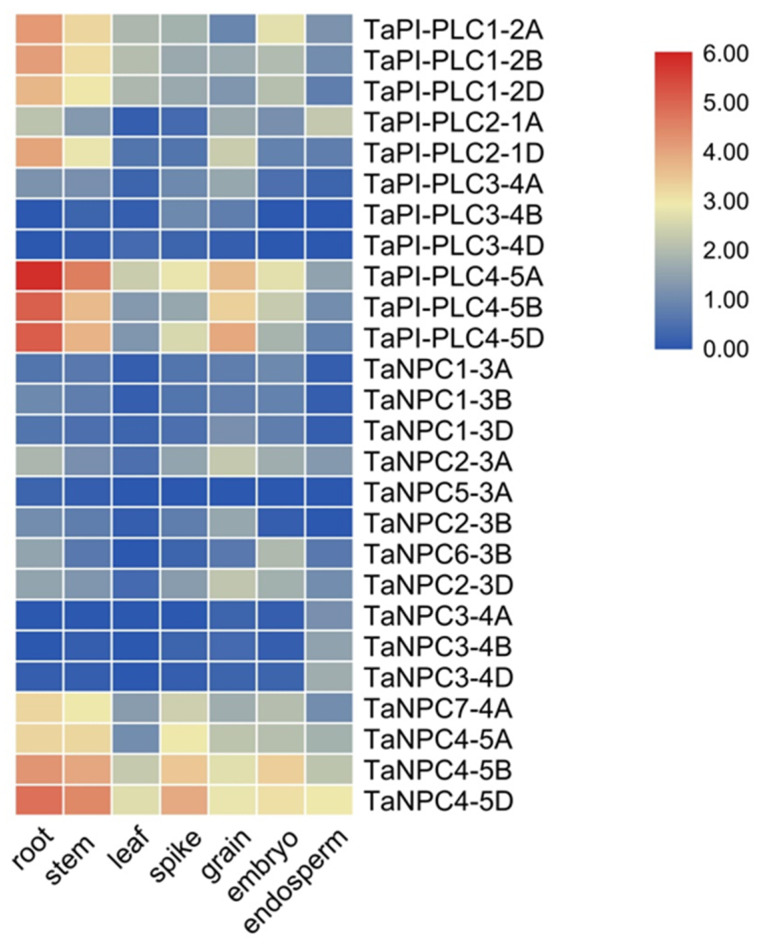
Expression profiles of *TaPLC* genes in 7 different tissues and organs (root, stem, leaf, spike, grain, embryo, endosperm). The data used in the figure were obtained from the wheat expression database (http://202.194.139.32/expression/index.html?tdsourcetag=s_pcqq_aiomsg).

The heatmap of 26 *TaPLC* genes was constructed by using RNA-seq data from the wheat expression database, and these genes were represented by TPM (Transcripts per million reads) values in seven different tissues and organs (root, stem, leaf, spike, grain, embryo, endosperm) (Figure 9). Within the *TaPI-PLC* subtype, only *TaPI-PLC3-4B* and *TaPI-PLC3-4D* were barely expressed in all tissues and organs, and the other 9 genes were significantly expressed in at least one tissue or organ. *TaPI-PLC1-2A*, *TaPI-PLC1-2B*, *TaPI-PLC1-2D*, and *TaPI-PLC2-1D* were highly expressed in roots and stems, while *TaPI-PLC4-5A*, *TaPI-PLC4-5B*, and *TaPI-PLC4-5D* were mainly expressed in roots, stems, and grains. Among *TaNPCs*, only *TaNPC7-4A*, *TaNPC4-5A*, *TaNPC4-5B*, and *TaNPC4-5D* had high expression levels, which were expressed in all seven tissues and organs. The heatmap results also showed that most homologous gene copies such as *TaPI-PLC1*, *TaPI-PLC4*, *TaNPC3*, and *TaNPC4* had similar expression patterns and were highly expressed in the same tissues. 

**Figure 10:**
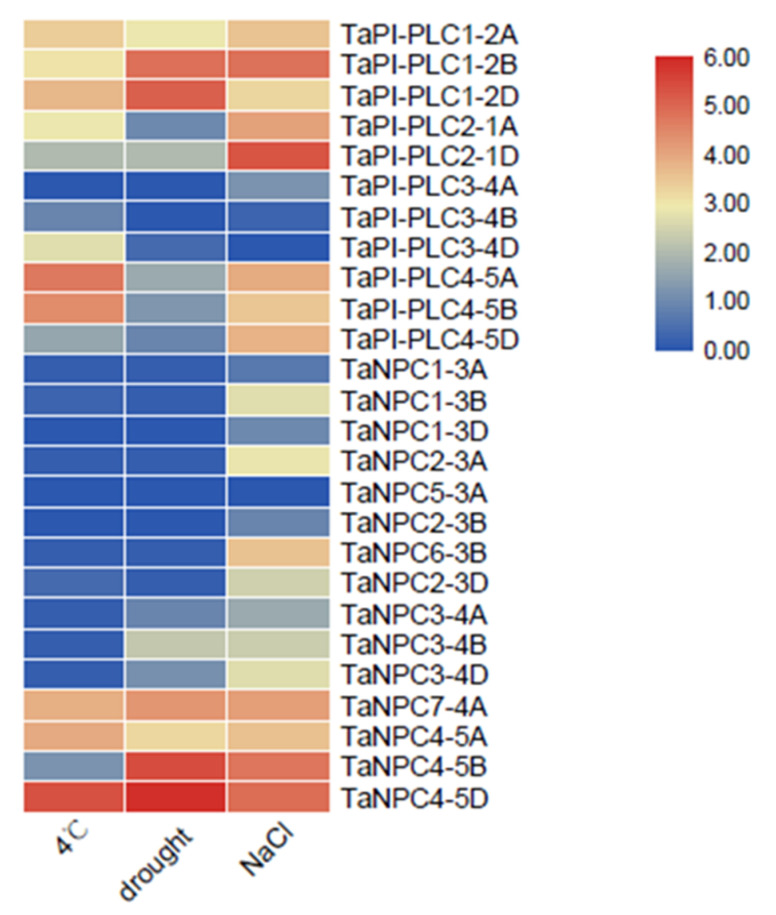
Expression profiles of 26 *TaPLC* genes under different stresses. The data used in the figure were obtained from the wheat expression database.

These results for gene expression under various abiotic stresses highlight the great importance of *PLC* family members in response to adverse conditions in plants (Figure 10). Under low temperature, drought, and salt stress, multiple copies of *TaPI-PLC1*, *TaPI-PLC2*, *TaPI-PLC3*, and *TaNPC4* were up-regulated in the PLC family of wheat. Conversely, *TaPI-PLC3-4A*, *TaPI-PLC3-4B*, *TaNPC1-3A*, *TaNPC1-3D*, and *TaNPC5-3A* were almost not expressed under the three abiotic stresses. Most *TaPLC* genes were expressed under salt stress, which indicated that members of the *PLC* gene family might be sensitive to salt.

### 2.7. Expression Profiles of TaPLCs in Leaves under Abiotic Stress

**Figure 11:**
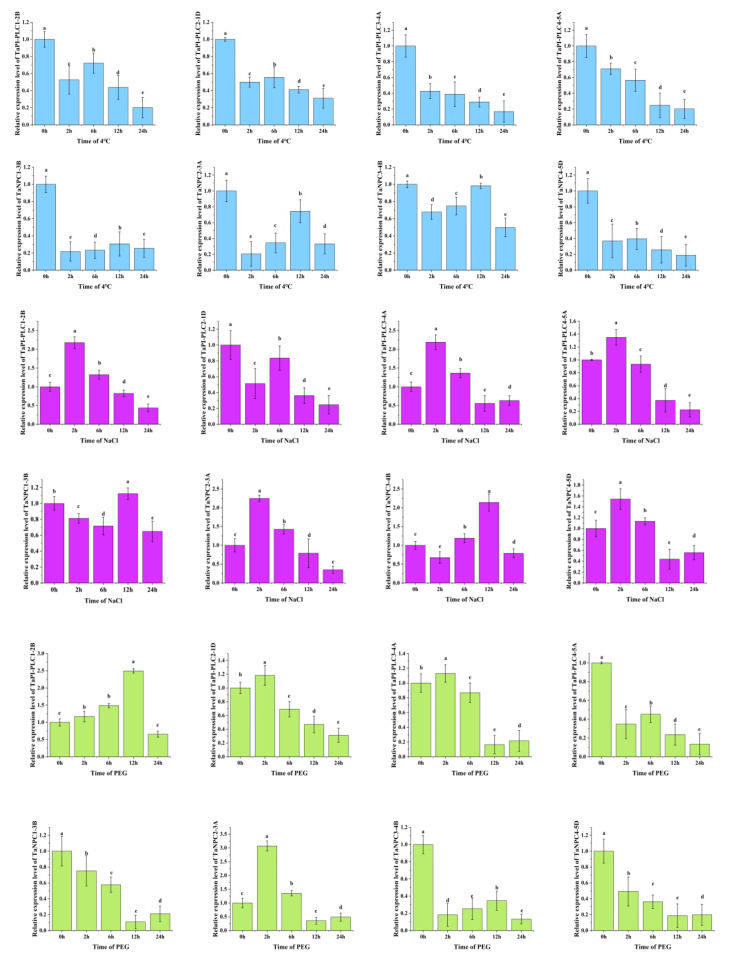
Gene expression profiles of *TaPLC* genes under drought (PEG) (green), salt (NaCl) (purple), and low temperature (4 °C) (blue) stress. The 2 ^-ΔΔCT^ method was used to calculate relative gene expression. In order to calculate the relative expression, the expression of each gene under the control treatment was set as 1. Error bars indicate standard deviations of three biological replicates. Different letters marked on the same bar chart indicate significant differences at the 0.05 level.

To further explore the expression changes in the *TaPLC* genes under various abiotic stresses including low temperature, salt, and drought, qRT-PCR was used to investigate the transcript levels of *TaPI-PLC1-2B*, *TaPI-PLC2-1D*, *TaPI-PLC3-4A*, *TaPI-PLC4-5D*, *TaNPC1-3B*, *TaNPC2-3A*, *TaNPC3-4B*, and *TaNPC4-5D* (Figure 11). 

Under drought stress, the expression levels of 8 *TaPLC* genes differed in leaves. The results showed that only the expressions of *TaPI-PLC1-2B*, *TaPI-PLC2-1D*, *TaPI-PLC3-4A*, and *TaNPC2-3A* were up-regulated after PEG treatment, while the others were significantly down-regulated. *TaPI-PLC2-1D*, *TaPI-PLC3-4A* and *TANPC2-3A* were firstly up-regulated, reached a peak at 2h, and then were down-regulated, while *TaPI-PLC1-2B* reached a peak at 12h. There was a high degree of agreement of *TaPI-PLC* between qPCR and public data, while data for *TaNPC* were quite different. 

Under low temperature stress at 4 °C, the expression levels of 8 *TaPLC* genes were significantly down-regulated. Interestingly, except for *TaPI-PLC3-4A* and *TaPI-PLC4-5A*, the other six genes showed only a slight upward and downward trend. The expression pattern of *TaNPC* was basically consistent with the results in Figure 10 and showed different expression pattern compares with *TaPI-PLCs*.

Under salt stress, *PLC* genes were significantly up-regulated except for *TaPI-PLC2-1D*. The expressions of *TaPI-PLC1-2B*, *TaPI-PLC3-4A*, *TaPI-PLC4-5A*, *TaNPC2-3A*, and *TaNPC4-5D* reached their peak values at 2h and then decreased, while the expressions of *TaNPC1-3B* and *TaNPC3-4B* did not reach peak values until 12h. The results were basically consistent with Figure 10, indicating that *PLC* genes were indeed sensitive to salt stress. Further, the different patterns of expression under different stress treatments indicated that members of the *TaPLC* gene family differ in their responses and regulatory mechanisms when exposed to conditions of abiotic stress.

### 2.8. Overexpression of TaPI-PLC1-2B Enhanced Abiotic Stress Resistance in Arabidopsis Transgenic Plants

**Figure 12:**
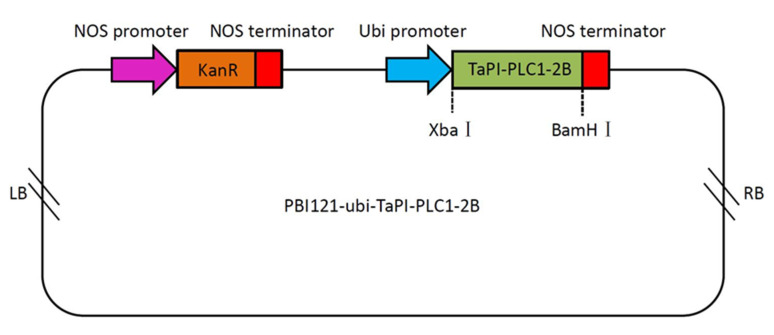
Schematic diagram of the overexpression vector containing TaPI-PLC1-2B. LB, left border; NOS promoter, nopaline synthase gene promoter; NOS terminator, nopaline synthase gene terminator; Ubi promoter, ubiquitin gene promoter; KanR, kanamycin resistance gene; RB, right border.

The *TaPI-PLC1-2B* gene is evolutionarily close to *OsPI-PLC1* in rice (Figure 4). In addition, the expression of *TaPI-PLC1-2B* was consistent with *OsPI-PLC1* under drought, salt and low temperature stress. Therefore, we hypothesized that *TaPI-PLC1-2B* might play an important role in abiotic stress response. In order to reveal the potential function of *TaPI-PLC1-2B*, an overexpression vector for it was constructed (Figure 12) and transgenic *Arabidopsis* plants were obtained.

**Figure 13:**
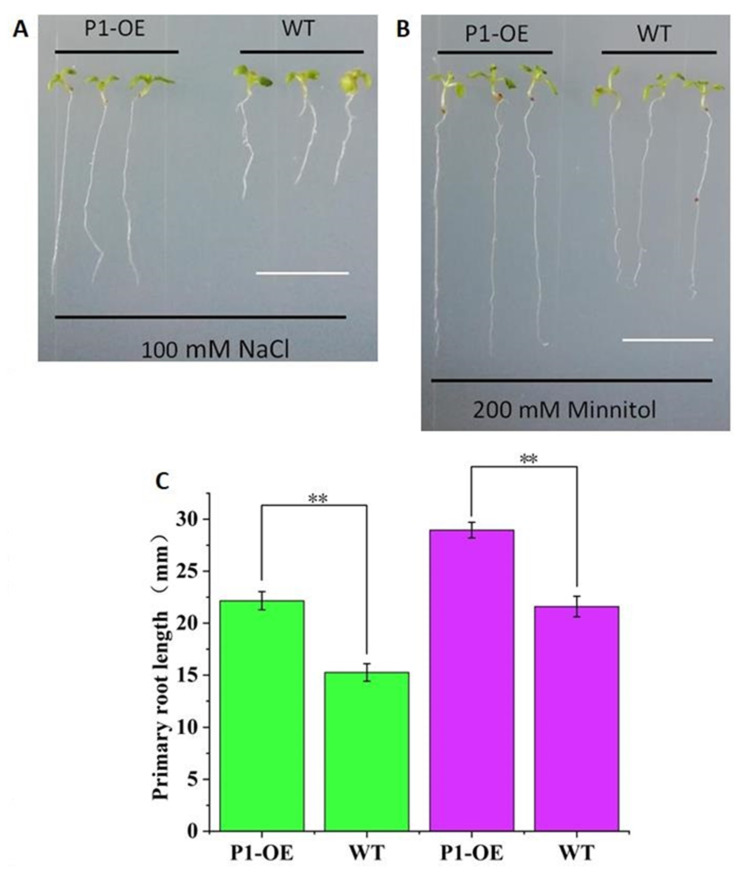
Analysis of the sensitivity of WT and *TaPI-PLC1-2B* overexpression (P1-OE) seedlings to NaCl and mannitol. (**A**) Phenotype of WT and *TaPI-PLC1-2B* transgenic seedlings grown on MS medium containing 100Mm NaCl for 10 days. (**B**) Ten-day-old WT and *TaPI-PLC1-2B* transgenic seedlings grown on MS medium containing 200 mM mannitol. (**C**) Primary roots lengths of the WT and *TaPI-PLC1-2B* transgenic plants at 10 day after sowing. The green rectangle represents NaCl treatment and the purple rectangle represents mannitol treatment. Bars = 10 mm. Mean values were calculated from 20 biological replicates, with error bars representing standard deviations. Statistically significant differences are indicated: **, *p* < 0.01.

The independent homozygous transgenic line (T3) was used for the experiments, and wild type (WT) *Arabidopsis* was used as the control. The *TaPI-PLC1-2B* transgenic seedlings (P1-OE) and the WT were planted on medium containing 100 mM NaCl and 200 mM mannitol. After 10 days of growth, the taproot lengths of P1-OE and WT plants were counted and photographed (Figure 13). The results showed that under the conditions of salt (100 mM NaCl) and drought (200 mM mannitol) stress, there were significant phenotypic differences between P1-OE and WT seedlings. On MS medium containing NaCl (Figure 13A, C) and mannitol (Figure 13B, C), the primary roots lengths of P1-OE seedlings were 31.15% and 25.39% longer than WT seedlings, respectively. These data indicated that overexpression of *TaPI-PLC1-2B* could significantly enhance salt and drought stress responses in *Arabidopsis*.

## 3. Discussion 

Improvements in sequencing and bioanalytical tools have opened the large genomes of crop plants to investigation, providing avenues to the identification of plant gene families and the mining of their functions [32,33]. This study used bioinformatics to identify a family of 26 *PLC* genes in wheat, a number greater than that in *Arabidopsis* (15), rice (9), and maize (9). These 26 genes had arisen through whole genome replication and tandem gene duplication events [34], and gene expansion also has enhanced the ability of wheat to adapt to complex and variable environments. However, there was considerable variation among the basic characteristics of wheat PLC proteins in features such as protein length, molecular weight, and isoelectric point. Due to the continuous insertion and deletion of introns, wheat *PLC* gene structure also has evolved considerably. Each member of the family contains from 1 to 8 introns and 2 to 9 exons, and some *PLC* genes contain UTR regions. Amino acid sequence analysis showed that all TaPI-PLCs have retained the three conserved X, Y and C2 domains, and only TaPI-PLC2-1A and TaPI-PLC2-1D have an EF-hand. All TaNPCs have phosphoesterase domains. The conserved amino acid sequences in the EF-hand and the X, Y, C2, and phosphoesterase domains may affect gene transcription and the activation and inhibition of wheat PLC proteins by binding or interacting with other factors. In the phylogenetic tree we constructed for the *Arabidopsis*, cotton, soybean, orchid, maize, rice, and wheat *PLC* gene families, the wheat, rice, and maize *PLC* genes were widely distributed within the same branch, indicating that they have higher homology, and the genetic relationship between wheat, rice, and maize *PLC* genes is closer than the relationship to *Arabidopsis*. Especially between wheat and rice, the relationship in Figure 7 showed syntenic *TaPLC* gene pairs between wheat and rice, and in combination with phylogenetic analysis, these results indicate that the *TaPLC* genes in wheat and rice share high homology. Further chromosome location analysis showed that the 26 *TaPLC* genes are located unevenly on 14 chromosomes, with most of them near the terminal regions. *TaPLC* genes were not found on chromosomes 6 and 7, which may be the result of genome-wide replication events during wheat evolution.

The expression patterns of *PLC* genes in different tissues have been described in different plant species such as *Arabidopsis*, rice, soybean, and cotton [3,5,15,35]. According to the RNAseq data provided by the database, expression patterns of the PLC genes in wheat were investigated in various tissues and organs. High transcript levels of *TaNPC4-5D* were detected in all tissues and organs, especially root and stem. *TaPI-PLC4-5A* showed the highest expression level in root, while *TaNPC2* and *TaPI-PLC8* showed low expression in various tissues and organs. A previous study showed *AtPLC2* transcripts were highly expressed in all organs in *Arabidopsis* [35], *GmPI-PLC7* were detected in all organs of cotton [3], *OsPLC1* and *OsPLC3* were highly expressed in all organs, but *OsPLC2* was detected in various organs with lower expression in rice [5]. The varied expression patterns of the wheat PLC genes imply that those genes may be involved in different stage or organ development of wheat.

Abiotic stress can induce a series of plant responses ranging from transcriptional regulation to signal transduction and the expression and activation of functionally specialized proteins [36]. Phospholipase genes play an important regulatory role in responding to environmental stress and because of their importance, members of the *PLC* gene family have been studied in considerable depth in other plants. For example, expression of all of the *Arabidopsis PI-PLCs* except for *AtPI-PLC2* was induced by abiotic stresses [5,35], and abscisic acid also induced up-regulation of *AtPI-PLC6* [24,37]. *AtPI-PLC6*, *AtPI-PLC7*, and *AtPI-PLC8* were up-regulated by auxin and cytokinin [9,31]. In other plants, the expression of mung bean *VrPI-PLC3*, tobacco *NtPI-PLC1*, potato *StPI-PLC1*, and *StPI-PLC2*, and tempeh bean *TluPI-PLC1* and *TluPI-PLC2* was influenced by drought stress [38,39,40,41]. In our study, the results of qRT-PCR showed that the *TaPLC* genes were responsive to drought, salt, and cold stress. Among the three abiotic stresses, the *TaPLC* genes were more sensitive to salt stress (Figure 10 and Figure 11).

Phylogenetic analysis suggests that proteins from different species belonging to the same evolutionary branch may have similar functions. For example, *OsNPC2* in rice was more sensitive to salt, and its expression level increased nearly eight times under salt stress [42]. The evolutionary relationship between *TaNPC2* and *OsNPC2* was relatively close (Figure 4 and Figure 11), and the expression of *TaNPC2-3A* increased by 2.5 times under salt stress. Similarly, *OsPI-PLC1* was simultaneously induced by various stresses and especially by salt treatment, and under salt stress its expression increased 2 times [43]. A study on maize showed that overexpression of a *ZmPI-PLC1* could improve drought tolerance of maize [44]. *TaPI-PLC1-2B* was highly homologous with *OsPI-PLC1 and ZmPI-PLC1,* and showed good resistance to both salt and drought stress, which was verified by the stress treatment of transgenic plants (Figure 13). Therefore, our study has shown that the expression of *TaPLC* genes changes under one or more abiotic stresses, indicating that they potentially have important roles in responses to environmentally adverse conditions.

## 4. Materials and Methods

### 4.1. Identification of PLC Family Members in the Wheat Genome

To identify potential *PLC* gene family members, we screened the entire protein sequence of the wheat genome from the PLAZA database (https://bioinformatics.psb.ugent.be/plaza/) with the HMM profiles (http://pfam.xfam.org/) PI-PLC-X (PF00388), PI-PLC-Y (PF00387), and PI-PLC-C2 (PF00168), the three domains characteristic of PI-PLC, and (PF04185) the domain characteristic of NPC proteins [5]. Those sequences with a cutoff value <0.001 and containing all three PLC-X, PLC-Y, and PLC-C2 domains were considered to be candidate members of the TaPI-PLC family, while those with only a phosphoesterase domain were designated potential TaNPC proteins. Putative PI-PLC protein sequences were submitted to CDD (https://www.ncbi.nlm.nih.gov/cdd), Pfam (http://pfam.xfam.org/), and SMART (http://smart.embl-heidelberg.de/) to confirm the presence of all three conserved domains.

### 4.2. Sequence Analysis and Structural Characterization of PLC Genes/Proteins in Wheat

PLC family member sequences were submitted to ExPASy (https://web.expasy.org/protparam/) to calculate the number of amino acids, molecular weight, theoretical isoelectric point (pI), and the grand average of hydrophobicity (GRAVY) of each protein [45]. MEME (http://meme-suite.org/tools/meme) was used to identify conserved domains with motifs = 10 [46]. The EvolView online tool (http://www.evolgenius.info/evolview/#login) was used to compare the predicted coding sequence (CDS) with the corresponding genomic sequence to detect the exon/intron distribution of the corresponding *TaPLC* genes [47]. 

### 4.3. Phylogenetic Distribution, Chromosome Location, and Gene Duplication Analyses

Phylogenetic analysis was performed using the protein sequences of rice, *Arabidopsis*, orchid, cotton, maize, and the newly identified TaPLCs (Table S1). MEGA7 software was used to perform multiple amino acid alignments of the sequences, with a bootstrap value of 1000 to construct a rootless phylogenetic tree by neighbor-joining (NJ) [48,49]. 

We used Map Gene 2 chromosome v 2 (MG2C) (http://mg2c.iask.in/mg2c_v2.0/?tdsourcetag=s_pcqq_aiomsg) to generate the map showing the position of the *TaPLC* genes in the chromosomes. To identify duplicated gene pairs, we defined gene duplication according to the following criteria [50]: (1) the alignable nucleotide sequence covered was >70% of the longer aligned gene, and (2) the aligned region had an identity >70%. The duplicated gene pairs were visualized using circle diagram. Non-synonymous (Ka) and synonymous (Ks) values were calculated by TBtools software [51].

### 4.4. Analysis of Cis-Acting Elements in TaPLC Genes’ Promoters

The upstream sequences (2000 bp) of the TaPLC coding sequences (CDS) were retrieved from PLAZA and submitted to PlantCARE (http://bioinformatics.psb.ugent.be/webtools/plantcare/html/) to identify *cis*-acting element. The Gene Structure Display Server (http://gsds.cbi.pku.edu.cn/) was used to draw diagrams [52].

### 4.5. RNA-Sequencing (RNA-seq) Data Analysis of PLC Genes

RNA-seq data for seven different abiotic stress, tissues, and organs (root, stem, leaf, spike, grain, embryo, endosperm) were obtained from the gene expression site of Chinese spring (http://202.194.139.32/expression/index.html?tdsourcetag=s_pcqq_aiomsg) and used to study the expression patterns of *TaPLC*s. Cluster analysis of the data was performed using TBtools software to generate heatmaps.

### 4.6. Plant Materials and Abiotic Stress Treatments

Common wheat (*Triticum aestivum* L., AABBDD, 2n = 6x = 42) Chinese spring (CS) was used as experimental material. Seeds with the same degree of fullness were selected for disinfection (70% alcohol for 1 min, 10% NaClO for 10 min, then washed by sterilized water 3 times). The sterilized seeds were placed on moistened filter paper in a sterile Petri dish and germinated in the dark for 48 h (25 °C). Seedlings were cultured with 16/8 h light, 25/20 °C temperature, and 70% relative humidity. The seedlings (15 days after germination) were cultured to two leaves. Salt stress was administered with 200mM sodium chloride (NaCl) and drought was simulated by treatment with 20% polyethylene glycol (PEG6000) [53]. The seedlings were incubated at 4 °C for low temperature stress. The leaves of the control and treatment groups were collected at 0, 2, 6, 12, and 24 h (Three duplicate samples were taken for each period.), immediately frozen with liquid nitrogen, and stored at −80 °C until RNA extraction.

### 4.7. Total RNA Extraction and Expression Analyses of Wheat PLC Genes

Oligo7 was used to design RT-PCR primers for *TaPLC* genes. Total RNA was extracted using the plant total RNA extraction kit (TIANGEN, Beijing, China). The cDNA was synthesized with a First Strand cDNA Synthesis Kit (TIANGEN, Beijing, China). 18S RNA was used as the internal reference gene [28], and the specific primer sequences of each gene are shown in Table S4. Reactions of 20 µL contained 2× Super-Real Mix 10 µL, 50× ROXII; 2 µL, forward and reverse primers 1 µl for each, cDNA 2µL, and ddH_2_O 4 µL. Reaction conditions included pre-denaturation at 95 °C for 15 min, denaturation at 95 °C for 10 s, annealing at 60 °C for 32 s, and 40 cycles. Three biological replicates and three technical replicates were applied for all qPCR analyses in this study. The 2^-ΔΔCT^ method was used to calculate relative gene expression. Software SPSS 19 was used for significance analysis and standard deviation calculation.

### 4.8. Generation of Transgenic Plants

The *TaPI-PLC1-2B* CDS was amplified through PCR using primers TaPI-PLC1-2B-C, and subsequently cloned into the PBI121 plant expression vector harboring the ubi promoter. *Arabidopsis* Columbia-0 was used to generate *TaPI-PLC1-2B* transgenic plants. Seedlings were cultured on half-strength Murashige and Skoog (MS) medium with 16/8 h light, 22/20 °C temperature, and 70% relative humidity. Salt stress was treated with 200 mM NaCl and drought was simulated by treatment with 200 mM mannitol [54]. Mannitol and NaCl were added directly to the MS medium. The transformation of the obtained recombinant vector into wild type (WT) *Arabidopsis* was performed via *an Agrobacterium tumefaciens* (Strain GV3101) mediated floral-dip method [55]. The transgenic plants were screened by using 50mg/L kanamycin [56].

## 5. Conclusions

In this study, we successfully performed a genome-wide analysis of the *PLC* family genes in wheat. 26 *TaPLC* genes in two subfamilies (*TaPI-PLC* and *TaNPC*) were identified in the genome of wheat. Both *PI-PLC* and *NPC* gene sequences showed high conservation as well as significant differences in wheat. Moreover, the 26 wheat *PLC* genes had different types and numbers of *cis*-regulatory elements in their respective promoters, consistent with their unique expression patterns in specific tissues and under different abiotic stresses. Through the analysis of the expression levels of some *TaPLC* gene family members under salt, drought, and low temperature stress, it was found that the PLC family members responded to the three abiotic stresses, especially salt stress. Furthermore, the *TaPI-PLC1-2B* gene was further studied and its overexpression vector was constructed and transferred to *Arabidopsis* for functional verification. The results showed that the *TaPI-PLC1-2B* transgenic seedlings had significantly improved resistance to salt stress and drought stress, and the primary root of P1-OE was significantly longer than that of the wild type. These results provide a solid basis for further investigation of biological functions of wheat *PLC* genes.

## Figures and Tables

**Table 1:** Features of *TaPLC* genes identified in wheat.

Gene Name	Transcript ID	Family	Exon no.	Location	Protein (AA)	MW (kDa)	PI	GRAVY
*TaPI-PLC1-2A*	TraesCS2A02G084000	PI-PLC	8	2A:38534860-38538480	608	68.4	5.91	−0.576
*TaPI-PLC1-2B*	TraesCS2B02G098500	PI-PLC	8	2B:58321242-58325230	606	68.2	6.06	−0.590
*TaPI-PLC2-2D*	TraesCS2D02G082000	PI-PLC	8	2D:35259471-35263395	607	68.5	5.88	−0.581
*TaPI-PLC2-1A*	TraesCS1A02G069300	PI-PLC	9	1A:51700021-51707185	585	66.0	6.07	−0.462
*TaPI-PLC1-1D*	TraesCS1D02G071800	PI-PLC	9	1D:52338417-52345805	586	66.2	6.03	−0.470
*TaPI-PLC3-4A*	TraesCS4A02G109000	PI-PLC	9	4A:129086595-129090166	633	71.1	5.54	−0.446
*TaPI-PLC3-4B*	TraesCS4B02G195200	PI-PLC	9	4B:420197892-420201623	633	71.0	5.75	−0.425
*TaPI-PLC3-4D*	TraesCS4D02G195800	PI-PLC	9	4D:340085748-340090648	633	71.1	5.84	−0.434
*TaPI-PLC4-5A*	TraesCS5A02G155300	PI-PLC	9	5A:333407514-333413175	590	65.7	6.05	−0.443
*TaPI-PLC4-5B*	TraesCS5B02G153600	PI-PLC	9	5B:283008744-283014326	589	65.7	6.06	−0.425
*TaPI-PLC4-5D*	TraesCS5D02G160300	PI-PLC	9	5D:250061407-250066699	589	65.7	6.06	−0.437
*TaNPC1-3A*	TraesCS3A02G083200	NPC	2	3A:53559328-53561696	531	58.4	7.81	−0.199
*TaNPC1-3B*	TraesCS3B02G098100	NPC	2	3B:65552302-65554643	531	58.6	7.26	−0.239
*TaNPC1-3D*	TraesCS3D02G083000	NPC	2	3D:42093593-42095880	531	58.3	8.32	−0.202
*TaNPC2-3A*	TraesCS3A02G492100	NPC	4	3A:719301726-719305850	533	60.0	5.98	−0.408
*TaNPC2-3B*	TraesCS3B02G552800	NPC	4	3B:787478591-787485906	530	58.6	5.94	−0.387
*TaNPC2-3D*	TraesCS3D02G499600	NPC	4	3D:589374117-589378796	537	59.4	6.07	−0.385
*TaNPC3-4A*	TraesCS4A02G298300	NPC	2	4A:597023152-597025088	513	56.7	6.05	−0.347
*TaNPC3-4B*	TraesCS4B02G015300	NPC	2	4B:11567063-11569283	516	57.1	5.95	−0.358
*TaNPC3-4D*	TraesCS4D02G013500	NPC	2	4D:6291692-6293849	514	57.0	5.88	−0.364
*TaNPC4-5A*	TraesCS5A02G489800	NPC	3	5A:659456675-659460043	540	60.0	7.01	−0.362
*TaNPC4-5B*	TraesCS5B02G503200	NPC	3	5B:669895600-669898631	542	60.2	7.31	−0.354
*TaNPC4-5D*	TraesCS5D02G504100	NPC	3	5D:530645503-530649282	541	60.1	7.01	−0.352
*TaNPC5-3A*	TraesCS3A02G439600	NPC	3	3A:682746317-682754957	554	61.7	7.13	−0.309
*TaNPC6-3B*	TraesCS3B02G553200	NPC	4	3B:787850236-787854279	530	58.6	5.98	−0.382
*TaNPC7-4A*	TraesCS4A02G369500	NPC	3	4A:641504088-641508259	540	60.1	7.01	−0.383

## Supplementary Materials

The following are available online at https://www.mdpi.com/2223-7747/9/7/885/s1, Figure S1: The *TaPLC* families motifs, Table S1: The IDs of *PLC* genes from Arabidopsis, soybean, rice, cotton, maize and Orchid, Table S2: The homologous *TaPLC* genes in wheat A, B and D sub-genomes and the Duplicated genes pairs identified in wheat, Table S3: Orthologous *PLC* gene pairs between wheat and rice, Table S4: Specific primers used for qRT-PCR analysis.
