# Supplementary material for: Genome-Wide Identification and Expression Profile Analysis of the Phospholipase C Gene Family in Wheat (Triticum aestivum L.)
Source: Plants (Basel). 2020 Jul 13;9(7):885. doi: 10.3390/plants9070885 (PMC7412115; doi:10.3390/plants9070885)
Supplement: Supplementary file 1 [file plants-09-00885-s001.pdf]

Table S1 The IDs of *PLC* genes from *Arabidopsis*, soybean, rice, cotton, maize and Orchid

Species	Gene name	Gene ID	Species	Gene name	Gene ID
	AtPI-PLC1	AT5G58670.1		GhPI-PLC1	CotAD_563
	AtPI-PLC2	AT3G08510.1		GhPI-PLC2	CotAD_094
	AtPI-PLC3	AT4G38530.1		GhPI-PLC3	CotAD_094
	AtPI-PLC4	AT5G58700.1		GhPI-PLC4	CotAD_094
	AtPI-PLC5	AT5G58690.1		GhPI-PLC5	CotAD_621
	AtPI-PLC6	AT2G40116.1		GhPI-PLC6	CotAD_302
	AtPI-PLC7	AT3G55940.1		GhPI-PLC7	CotAD_185
Arabidopsis	AtPI-PLC8	AT3G47290.1		GhPI-PLC8	CotAD_185
	AtPI-PLC9	AT3G47220.1		GhPI-PLC9	CotAD_185
	AtNPC1	AT1G07230.1		GhPI-PLC10	CotAD_22
	AtNPC2	AT2G26870.1	Cotton	GhPI-PLC11	CotAD_22
	AtNPC3	AT3G03520.1		GhPI-PLC12	CotAD_22
	AtNPC4	AT3G03530.1		GhNPC1	CotAD_60511
	AtNPC5	AT3G03540.1		GhNPC2	CotAD_72613
	AtNPC6	AT3G48610.1		GhNPC3	CotAD_58558
	OsPI-PLC1	LOC_Os07g49330.1		GhNPC4	CotAD_16432
	OsPI-PLC2	LOC_Os03g18010.1		GhNPC5	CotAD_59323
	OsPI-PLC3	LOC_Os12g37560.1		GhNPC6	CotAD_03840
	OsPI-PLC4	LOC_Os05g03610.1		GhNPC7	CotAD_27559
Rice	OsNPC1	LOC_Os03g61130.1		GhNPC8	CotAD_70096
	OsNPC2	LOC_Os01g72520.1		GhNPC9	CotAD_39172
	OsNPC3	LOC_Os11g38050.1		ZmPI-PLC1	GRMZM2G114354-T01
	OsNPC4	LOC_Os03g63580.1		ZmPI-PLC2	GRMZM2G129238-T01
	OsNPC5	LOC_Os01g01190.1		ZmPI-PLC3	GRMZM2G137435-T01
	PePI-PLC1	PEQU_10585		ZmPI-PLC4	GRMZM5G889467-T01
Orchid	PePI-PLC2	PEQU_11930	Maize	ZmPI-PLC5	GRMZM2G157760-T01
	PePI-PLC3	PEQU_14284		ZmNPC1	GRMZM2G081719-T01
	GmPI-PLC1	AAA74441.1		ZmNPC2	GRMZM2G479112-T01
	GmPI-PLC2	XP_003552353.1		ZmNPC3	GRMZM2G139041-T01
	GmPI-PLC12	XP_003538468.2		ZmNPC4	GRMZM2G422670-T01
Soybean	GmPI-PLCp12	AAB03257.1			
	GmPI-PLCp13	AAB03258.1			
	GmPI-PLCp25	AAB03259.1			

Table S2 The homologous *TaPLC* genes in wheat A, B and D sub-genomes and the Duplicated genes pairs identified in wheat

Gene ID	Genomic location		Gene ID	Genomic location		Ka/Ks
	Start	End		Start	End	
TaPI-PLC2-1A	51700021	51707185	TaPI-PLC2-1D	52338417	52345805	0.1330
TaPI-PLC1-2A	38534860	38538480	TaPI-PLC1-2B	58321242	58325230	0.0906
TaPI-PLC1-2A	38534860	38538480	TaPI-PLC1-2D	35259471	35263395	0.0934
TaPI-PLC1-2B	58321242	58325230	TaPI-PLC1-2D	35259471	35263395	0.1080
TaPI-PLC1-2D	35259471	35263395	TaPI-PLC3-4A	129086595	129090166	0.1886
TaPI-PLC1-2D	35259471	35263395	TaPI-PLC3-4B	420197892	420201623	0.1729
TaPI-PLC1-2D	35259471	35263395	TaPI-PLC3-4D	340085748	340090648	0.1749
TaPI-PLC3-4A	129086595	129090166	TaPI-PLC3-4B	420197892	420201623	0.2360
TaPI-PLC3-4A	129086595	129090166	TaPI-PLC3-4D	340085748	340090648	0.1541
TaPI-PLC3-4B	420197892	420201623	TaPI-PLC3-4D	340085748	340090648	0.2015
TaPI-PLC4-5A	659456675	659460043	TaPI-PLC4-5B	669895600	669898631	0.0466
TaPI-PLC4-5A	659456675	659460043	TaPI-PLC4-5D	530645503	530649282	0.0412
TaPI-PLC4-5B	669895600	669898631	TaPI-PLC4-5D	530645503	530649282	0.0417
TaNPC1-3A	53559328	53561696	TaNPC1-3B	65552302	65554643	0.1763
TaNPC1-3A	53559328	53561696	TaNPC1-3D	42093593	42095880	0.1135
TaNPC2-3A	719301726	719305850	TaNPC2-3D	589374117	589378796	0.0692
TaNPC2-3B	787478591	787485906	TaNPC2-3D	589374117	589378796	0.0880
TaNPC3-4A	597023152	597025088	TaNPC3-4B	11567063	11569283	0.0795
TaNPC3-4A	597023152	597025088	TaNPC3-4D	6291692	6293849	0.0774
TaNPC3-4B	11567063	11569283	TaNPC3-4D	6291692	6293849	0.1072
TaNPC4-5A	659456675	659460043	TaNPC4-5B	669895600	669898631	0.0780
TaNPC4-5A	659456675	659460043	TaNPC4-5D	530645503	530649282	0.0853
TaNPC4-5B	669895600	669898631	TaNPC4-5D	530645503	530649282	0.0652
TaNPC7-4A	641504088	641508259	TaNPC4-5B	669895600	669898631	0.0342
TaNPC7-4A	641504088	641508259	TaNPC4-5D	530645503	530649282	0.0549

Table S3 Orthologous PLC gene pairs between wheat and rice

Gene ID	Chr.	Genomic location		Gene ID	Chr.	Genomic location		Ka/Ks
		Start	End			Start	End	
TaPI-PLC1-2A	2A	38534860	38538480	OsPI-PLC1	7	29545825	29548981	0.2377
TaPI-PLC1-2B	2B	58321242	58325230	OsPI-PLC1	7	29545825	29548981	0.2514
TaPI-PLC1-2D	2D	35259471	35263395	OsPI-PLC1	7	29545825	29548981	0.2180
TaPI-PLC1-2A	2A	38534860	38538480	OsPI-PLC2	3	10025401	10027931	0.1350
TaPI-PLC1-2B	2B	58321242	58325230	OsPI-PLC2	3	10025401	10027931	0.1265
TaPI-PLC1-2D	2D	35259471	35263395	OsPI-PLC2	3	10025401	10027931	0.1408
TaPI-PLC3-4A	4A	129086595	129090166	OsPI-PLC1	7	29545825	29548981	0.1633
TaPI-PLC3-4B	4B	420197892	420201623	OsPI-PLC1	7	29545825	29548981	0.1698
TaPI-PLC3-4D	4D	340085748	340090648	OsPI-PLC1	7	29545825	29548981	0.1703
TaPI-PLC3-4A	4A	129086595	129090166	OsPI-PLC2	3	10025401	10027931	0.1280

TaPI-PLC3-4B	4B	420197892	420201623	OsPI-PLC2	3	10025401	10027931	0.1237
TaPI-PLC3-4D	4D	340085748	340090648	OsPI-PLC2	3	10025401	10027931	0.1179
TaPI-PLC2-1A	1A	51700021	51707185	OsPI-PLC4	5	1535708	1541571	0.2181
TaPI-PLC2-1D	1D	52338417	52345805	OsPI-PLC4	5	1535708	1541571	0.2042
TaNPC1-3A	3A	53559328	53561696	OsNPC5	1	89653	91521	0.3355
TaNPC1-3B	3B	65552302	65554643	OsNPC5	1	89653	91521	0.3124
TaNPC1-3D	3D	42093593	42095880	OsNPC5	1	89653	91521	0.3078
TaNPC2-3A	3A	719301726	719305850	OsNPC2	1	42063181	42067788	0.2089
TaNPC2-3D	3D	589374117	589378796	OsNPC2	1	42063181	42067788	0.1840
TaNPC4-5A	5A	659456675	659460043	OsNPC1	3	34724320	34727310	0.1337
TaNPC4-5B	5B	669895600	669898631	OsNPC1	3	34724320	34727310	0.1351
TaNPC4-5D	5D	530645503	530649282	OsNPC1	3	34724320	34727310	0.1534
TaNPC7-4A	4A	641504088	641508259	OsNPC1	3	34724320	34727310	0.1500

Table S4 Specific primers used for qRT-PCR analysis

Gene	Forward Primer (5'-3')	Reverse Primer (5'-3')
TaPI-PLC1-2B-C	CTCCAAACTTCCAAAGCCATG	TCACACAAACTCAAAGCGCAT
TaPI-PLC1-2B-RT	GGGCACTCGGGTTACTTC	GTAGCCACAGCCACCATT
TaPI-PLC2-1D-RT	CAACGCGCATTACTTCGTCCA	GTCGAATCGCCATCCGTCT
TaPI-PLC3-4A-RT	TAAAGCAGGAAAGCCGAAGG	ACCATGCACCCAGCCAAG
TaPI-PLC4-5A-RT	ATCACGGAAACATTTGGAGACC	TGTGGTGGTTTAGTTGAGATGA
TaNPC1-3B-RT	CCTGCTCTCCTCCGCCGTCAT	AGTAGAGGAAGAGGCGGTTGG
TaNPC2-3A-RT	AGGTTGGGAGTGCGTGTCC	TGTCCTCGGTTCAGTTCTGGTTT
TaNPC3-4B-RT	ACATGCTGGGCTGGATGAAAT	TGCCGTAGACCTGCTCGTAGATAG
TaNPC4-5D-RT	GGTTTGTCAAGGAGGTCTACGA	CCCATCAGGCTGAGGCACT
18sRNA	GCATTTGCCAAGGATGTTTTC	TGCTATGTCTGGACCTGGTAAGT

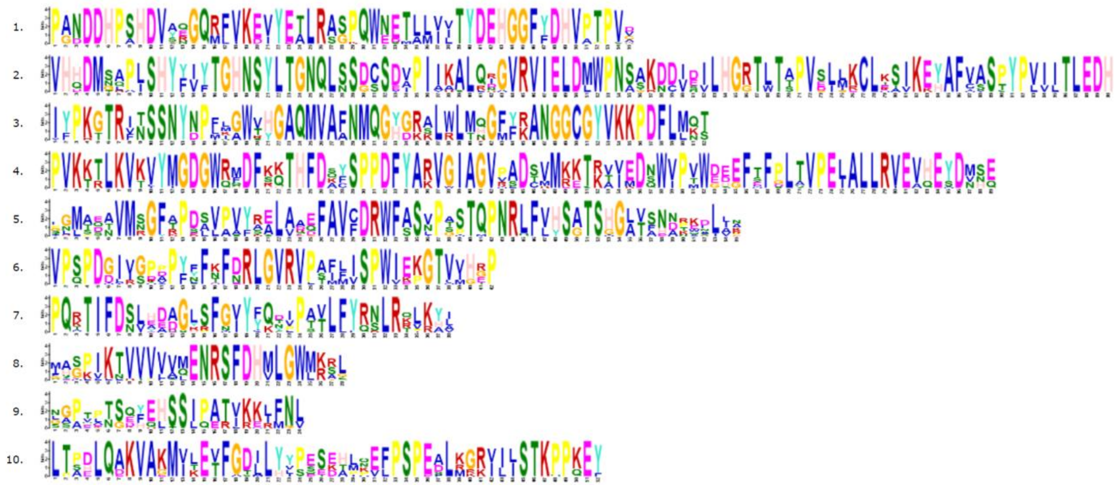

Fig. S1 The TaPLC families motifs. The overall height of each stack indicates the conservation of the sequence at that position, whereas the height of letters within each stack represents the relative frequency of the corresponding amino acid.
